# Near-Optimal Recovery Within 3 Months: Investigating Health-Related Quality of Life and Functional Outcomes After Single-Stage Bilateral Hip Replacement for Osteoarthritis

**DOI:** 10.1097/NOR.0000000000000987

**Published:** 2023-12-04

**Authors:** Daniël O. Strijbos, Tim A. E. J. Boymans, Richard Bimmel

**Affiliations:** **Daniël O. Strijbos, MSc,** PhD candidate, at Amsterdam UMC, Amsterdam, the Netherlands; Physiotherapist, Nij Smellinghe Hospital Drachten, Drachten, the Netherlands.; **Tim A. E. J. Boymans, MD, PhD,** Orthopedic surgeon, Maastricht UMC +, Maastricht, the Netherlands.; **Richard Bimmel, MD,** Orthopedic surgeon, Nij Smellinghe Hospital Drachten, Drachten, the Netherlands.

## Abstract

Single-stage bilateral hip replacement (SSBHR) is a safe and successful orthopaedic intervention for patients suffering from bilateral osteoarthritis of the hip. Data on short- and mid-term recovery outcome studies are, unfortunately, scarce. The purpose of this study was to investigate the change in the functional measures and quality of life after SSBHR and to determine the patient's willingness to undergo the same procedure again. Data were prospectively collected and analyzed from patients with bilateral symptomatic hip osteoarthritis who underwent SSBHR from January 2019 until December 2020. Patients were excluded only if they failed to sign an informed consent or were unable to fill out questionnaires due to language or cognitive problems. Preoperatively and 3 and 12 months after surgery, health-related quality of life (HRQOL) and physical functioning were measured. Twelve months after surgery, patient satisfaction (willingness to undergo the same procedure again) was obtained. Complications, blood loss, and length of stay (LOS) were abstracted from the clinical notes and the electronic patient files. Patients improved significantly on all domains of HRQOL (16.0%–59.7%) and physical functioning (14.7%–15.8%) 3 months after surgery in comparison with preoperatively. No improvement was reported on HRQOL and physical functioning, except the Timed Up and Go score (14.1%), at 12 months after surgery in comparison with 3 months. No major or minor complications were found, and LOS was 2.9 days on average. One year after the surgery, all patients expressed satisfaction as suggested by their willingness to undergo the same surgical procedure again. Our study demonstrates that SSBHR offers a rapid recovery time and significant improvements in both functional status and HRQOL within 3 months after surgery. These findings can inform healthcare professionals and patients, suggesting that SSBHR is a viable treatment option for patients with bilateral hip osteoarthritis. Further research, including multicenter randomized controlled trials, is recommended to compare the recovery outcomes of SSBHR with two-stage bilateral hip replacement and confirm our findings.

## Introduction

Total hip arthroplasty (THA) is one of the most successful orthopaedic interventions of modern times. The number of THA procedures are expected to increase 174% between 2005 and 2030 (from 328,735 to 572,000) in the United States; 192% from 2010 to 2035 (from 50,000 to 95,877) in the United Kingdom; 208% from 2003 to 2030 (from 25,945 to 79,795) in Austria; and 150% from 2005 to 2030 (from 13,785 to 31,731) in the Netherlands (Ackerman et al., 2019; Culliford et al., 2015; Kurtz et al., 2007; Otten et al., 2010). Almost 70% of these patients have radiographic bilateral osteoarthritis (OA) of the hip (Chitnavis et al., 2000). An estimated 20% of them will eventually end up with a bilateral total hip replacement (LROI, 2014).

The majority of bilateral hip replacements are performed in two stages (sequentially), leaving a variable amount of time, ranging from 1 week to more than 1 year, between operations. Another option is single-stage bilateral hip replacement (SSBHR). Expected benefits of SSBHR for patients are less stress due to one major life event (undergoing surgery) instead of two, shorter rehabilitation time, and possibly a shorter dependency in activities of daily living. Benefits also include undergoing anesthesia once, only one hospital admission and therefore overall shorter length of stay (LOS), and cost reduction (Alfaro-Adrián et al., 1999; Parvizi et al., 2006; Reuben et al., 1998).

The safety of using the single-stage bilateral procedure versus staged hip replacement surgery has been studied extensively. Most research was done before the fast-track era, which was implemented in our hospital starting 2013. Fast-track surgery is a coordinated effort, combining modern concepts of patient education with newer anesthetic and analgesic methods and minimally invasive surgical techniques, the intention of which was to reduce the stress response, and minimize pain and discomfort (Kehlet, 2015). Most studies examining SSBHR focused on perioperative complication rates. A 2017 meta-analysis found no significant difference in death, pulmonary embolism, cardiovascular complications, infection, minor complications, and other surgical complications for SSBHR in comparison with staged hip replacement (Shao et al., 2017). A 2018 systematic review concluded that patients 65 years and younger without cardiovascular comorbidities should preferably undergo SSBHR instead of two-staged THA, although the available evidence is heterogeneous and the quality of evidence is low.

Nowadays, advances in surgical technique, blood management before and after surgery, and the start of prehabilitation programs (such as Better in, Better out) opened new interest in SSBHR (Hulzebos & and van Meeteren, 2016). It was at this time that SSBHR was introduced as usual care in a regional hospital in the northern part of the Netherlands in 2018.

Short- and mid-term rehabilitation outcomes can be described as changes in quality of life (QOL) and functional outcomes (Lee et al., 2014). Data on QOL and functional outcomes after SSBHR are, unfortunately, scarce. Our aim was to gain insight into recovery after SSBHR by investigating the change in preoperative functional measurements and QOL in comparison with 3 and 12 months after surgery. At the 12-month postsurgery mark, patients were also asked whether they would opt for the same surgery again given the choice.

## Materials and Methods

### Study Design and Participants

In this single-center, prospective, observational cohort study (Evidence Level 2), data were collected and analyzed from a sample of patients with bilateral symptomatic hip OA who underwent single-stage bilateral THA from January 2019 until December 2020. During this period, all patients with bilateral symptomatic hip OA were offered SSBHR. Only one patient decided to go with a staged procedure, as they were fearful that the rehabilitation period for the bilateral procedure would be too intense. There were no medical reasons encountered that precluded offering SSBHR. Patients were excluded only if they failed to sign an informed consent or were unable to fill out questionnaires due to language or cognitive problems.

This study was conducted in accordance with the declaration of Helsinki and Directive 95/26/EG of the European Union regarding data protection. According to Dutch law, research with anonymized care data acquired by the healthcare professional treating the patient does not require approval from a medical ethical committee. Patients who participated in this study were asked to sign an informed consent to analyze and use their medical data for research purposes. This was confirmed by the ethical committee of the regional hospital with respect to this particular study (Reference ID: 12051).

### Setting

All surgical procedures were performed at a regional community hospital situated in the northern region of the Netherlands. Within this facility, patients scheduled for arthroplasty surgery are subject to preoperative assessments (Better in, Better out) conducted by an in-house physical therapist (Bongers et al., 2016). This screening process employs validated tests to determine each patient's functional status. When deemed necessary, patients participate in a standard home-based prehabilitation program. The primary indications for hip replacement included significant bilateral symptomatic hip OA or mild dysplastic osteoarthritic deformed hips.

Joint replacement surgical procedures were performed in the lateral decubitus position, utilizing a posterior piriformis-sparing approach and adhering to contemporary fast-track principles (Altman et al., 2019). This approach optimizes patient care, enabling mobilization within 4 hours postsurgery, facilitated through spinal anesthesia administration and intravenous tranexamic acid pre- and postoperation, barring any contraindications. Postoperative physical therapy commenced as spinal anesthesia effects subsided.

The nursing care for SSBHR and unilateral THR care are essentially the same. Nursing care prioritizes equipping patients with the necessary skills for a safe recovery at home, self-administering pain relief medication, and monitoring vitals. At Nij Smellinghe Hospital in Drachten, the standard approach for mobility incorporates a multidisciplinary collaboration between nursing and physiotherapeutic care to achieve functional recovery within the hospital after surgery. The physical therapist assesses the patient's required assistance level for transfers and mobilization, as well as their capacity to handle physical strain. The nurse then facilitates functional movement exercises based on these assessments. Discharge criteria include the following factors: satisfactory functional outcomes (modified Iowa Level of Assistance scores at either 0, indicating no assistance needed, or 6, which signifies that testing was not necessary or applicable to the patient's situation), acceptable pain levels, absence of significant wound leakage, and stable vital parameter measurements (Elings et al., 2019).

### Recovery Outcomes

To measure recovery after single-stage bilateral THA, health-related quality of life (HRQOL) and functional outcomes were measured before surgery (*T*_0_) and 3 (*T*_1_) and 12 months (*T*_2_) after surgery (Lee et al., 2014). Twelve months postoperatively, patients were asked whether they would choose SSBHR again or not. HRQOL was measured with the short form survey (SF-36, also known as RAND-36). Functional outcomes were measured with the Timed Up and Go (TUG) test and the de Morton Mobility Index (DEMMI), both of which are a measure of functional recovery (van der Sluis et al., 2017).

The SF-36 is a self-report HRQOL questionnaire containing 36 questions in nine different domains. These domains are physical functioning (PF), social functioning (SF), role physical (RP), role emotional (RE), mental health (MH), vitality (VT), bodily pain (BP), general health (GH), and health change (HC). Answers to these questions are added up to a raw score within every domain. This raw score can be converted to a 0–100 scale score. The SF-36 has demonstrated reliability, validity, and responsiveness to change in patients with OA (Ware & Sherbourne, 1992). For this study, participants used the validated Dutch translation of the SF-36 (mean Cronbach α coefficient of 0.84) (Aaronson et al., 1998).

The DEMMI is a performance-based test to assess the degree of mobility of general hospitalized patients specifically in situation of transfer, static and dynamic balance, and walking. It consists of 15 items leading to a total score ranging from 0 to 100, with a higher score indicating better functional mobility (de Morton et al., 2008). The Dutch translation of the DEMMI has an intraclass correlation coefficient (ICC) for interrater reliability (IRR) of .85 and a minimal clinical important difference (MCID) of 7 points in older patients suffering from OA (Jans et al., 2011).

The TUG test is a performance-based test to assess a person's mobility. The TUG test measures the time it takes for a person to stand up from a chair, walk 3 m, turn around, walk back to sit in the chair again. The TUG test has an ICC for IRR of .96 for patients with OA Grades 1–3 (Alghadir et al., 2015). The TUG test has an MCID of −1.20 for patients with hip OA (Wright et al., 2011).

### Data Analysis

Data were analyzed using SPSS 26.0. Patients' characteristics were analyzed using mean and standard deviation or frequency for numeric variables and percentage for categorical variables. Differences in HRQOL and functional variables (*T*_0_ vs. *T*_1_, *T*_1_ vs. *T*_2_, and *T*_0_ vs. *T*_2_) were analyzed using a one-tailed dependent (paired) *t* test. Statistical significance level was set at *p* < .05.

## Results

**Figure 1.**
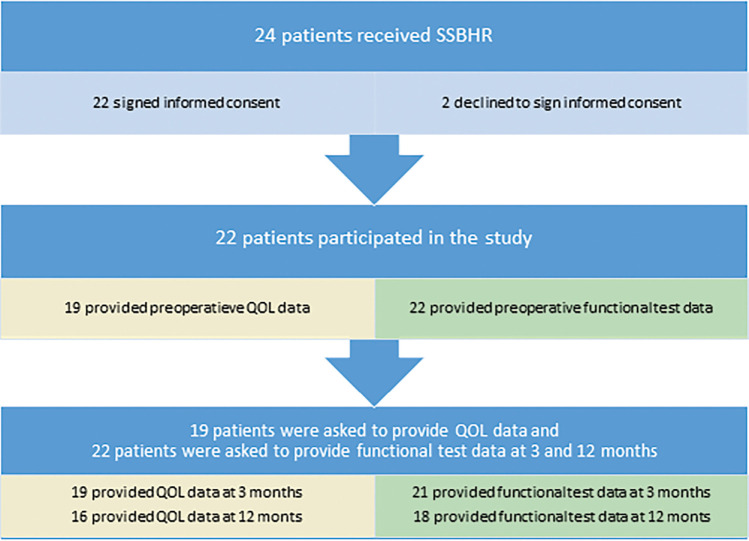
The flowchart illustrating the participants included in the study and the type of data collected. Yellow boxes represent data collected on quality of life, whereas green boxes represent data collected on functional measurements. The color versions of all figures are available in the online issue at https://journals.lww.com/orthopaedicnursing. QOL = quality of life; SSBHR = single-stage bilateral hip replacement.

In total, 22 of 24 patients who received SSBHR between January 2019 and December 2020 participated in this study. The two patients who did not participate did not sign the informed consent due to perceived time effort and were excluded. Table 1 shows the baseline characteristics of the participants. The mean age of the patients was 69.6 years. The majority of SSBHR procedures were performed in females (72.7%). The LOS was 2.9 days including the day of surgery. Three patients did not provide QOL data preoperatively (see Figure 1).

**Table 1.** Characteristics and Descriptive Statistics of the Study Sample

Characteristics	Number (% Missing)	Mean (*SD*)/Frequency (%)
*Demographic variables*		
Age	22 (0)	69.6 (7.4)
Sex	22 (0)	
Female		16 (72.7)
ISAR	22 (0)	0.6 (0.7)
BMI (kg/m^2^)	22 (0)	29.32 (3.7)
ASA	22 (0)	2.0 (0.4)
*Health-related quality of life preoperatively*	19 (13.6)	
Physical functioning		41.7 (23.5)
Social functioning		63.2 (29.9)
Physical role functioning		13.9 (27.4)
Emotional role functioning		53.7 (50.0)
Mental health		64.0 (21.8)
Vitality		51.7 (16.0)
Bodily pain		56.5 (21.5)
General health perception		63.8 (17.8)
Change in health		23.6 (21.8)
*Functional variables preoperatively*		
TUG (seconds)	22 (0)	10.1 (3.9)
DEMMI	22 (0)	80.4 (14.9)
*Surgical related variables*		
Blood loss during surgery (ml)	19 (13.6)	384.2 (134.4)
Time of operation (minutes)	22 (0)	116 (11.5)
Length of stay (days)	22 (0)	2.9 (1.1)
Complication	22 (0)	
Yes		0 (0)
Discharge location	22 (0)	
Home		22 (100)

*Note*. ASA = American Society of Anesthesiologists; BMI = body mass index; DEMMI = de Morton Mobility Index; ISAR = Iowa Senior At Risk; TUG = Timed Up and Go.

Throughout the follow-up period, all patients submitted QOL data. However, at the 12-month mark, three patients failed to provide this information. All patients, except one, participated in the follow-up functional assessments. At the 12-month follow-up, four patients were absent from the functional measurements (see Figure 1). One patient's absence was due to a terminal illness unrelated to SSBHR, whereas the others missed the appointment due to nonattendance at the clinic.

Patients improved on all HRQOL domains and all functional variables within 3 months after surgery in comparison with the similar preoperative variables (see Table 2). The mean improvement in QOL, in comparison with preoperatively, was 32.7% and the mean functional measurements improved, in comparison with preoperatively, 1.6 seconds on the TUG test and 11.8 points on the DEMMI 3 months after surgery. The biggest improvements were in the SF-36 domains physical role functioning (54.2% improvement) and change in health (59.7% improvement) in time.

**Table 2.** Health-Related Quality of Life and Functional Variables Before and After Surgery

Characteristics	Mean *T*_0_ (*SD*)	Mean *T*_1_ (*SD*)	Mean *T*_2_ (*SD*)	MD (*p*) [95% CI] *T*_1_–*T*_0_	MD (*p*) [95% CI] *T*_2_–*T*_0_	MD (*p*) [95% CI] *T*_2_–*T*_1_
Health-related quality of life						
Physical functioning	41.7 (23.6)	74.4 (17.8)	77.7 (17.9)	**32.8 (.000) [20.5, 45.0]**	**32.2 (.001) [15.0, 49.4]**	4.2 (.208) [6.7, 15.0]
Social functioning	63.2 (29.9)	86.7 (16.9)	91.3 (12.9)	**23.5 (.004) [6.8, 40.2]**	**27.8 (.007) [7.5, 48.0]**	5.2 (.159) [−5.7, 16.2]
Physical role functioning	13.9 (27.4)	68.1 (35.2)	69.2 (44.7)	**54.2 (.000) [30.3, 78.0]**	**52.8 (.005) [16.2, 89.3]**	−2.1 (.423) [−25.0, 20.8]
Emotional role functioning	53.7 (50.0)	77.8 (37.9)	84.4 (30.5)	**24.1 (.031) [**−**1.2, 49.4]**	22.2 (.076) [−9.5, 53.9]	2.2 (.360) [−10.8, 15.2]
Mental health	64.0 (21.8)	80.0 (13.1)	82.7 (12.8)	**16.0 (.004) [4.7, 27.3]**	**12.7 (.027) [**−**0.2, 25.5]**	1.1 (.351) [−4.8, 6.9]
Vitality	51.7 (16.0)	72.8 (12.2)	70.7 (15.0)	**21.1 (.000) [11.1, 31.1]**	**18.7 (.000) [9.6, 27.8]**	0.6 (.423) [−6.4, 5.3]
Bodily pain	36.5 (22.5)	77.1 (13.6)	76.7 (19.3)	**40.6 (.000) [28.8, 52.4]**	**42.1 (.000) [29.7, 54.5]**	−1.1 (.415) [−9.7, 11.9]
General health perception	63.9 (17.8)	86.1 (10.5)	84.7 (12.3)	**22.2 (.000) [11.8, 32.6]**	**23.0 (.000) [14.3, 31.7]**	−0.6 (.417) [−6.1, 5.0]
Change in health	23.6 (21.8)	83.3 (21.0)	83.3 (24.3)	**59.7 (.000) [44.3, 75.2]**	**56.7 (.000) [34.2, 79.2)**	0 (.500) [−16.5, 16.5]
Functional variables						
TUG (seconds)	10.1 (4.0)	8.5 (2.5)	7.3 (1.9)	−**1.6 (.009) [**−**2.9,**−**0.3]**	−**2.4 (.000) [**−**3.4,**−**1.4,]**	−**1.2 (.000) [**−**1.9,**−**0.66]**
DEMMI	80.2 (15.3)	92.0 (10.1)	94.4 (8.5)	**11.8 (.000) [6.1, 17.5]**	**15.2 (.001) [7.6, 22.8]**	2.2 (.149) [−2.2, 6.7]

*Note*. DEMMI = de Morton Mobility Index; MD = mean difference; TUG = Timed Up and Go; *T*_0_= preoperative; *T*_1_= 3 months postoperatively; T2 = 12 months postoperatively. **Bold** indicates significant *p* value.

Twelve months after surgery, most patients did not statistically improve on any of the domains of HRQOL or on the DEMMI, in comparison with 3 months after surgery. The TUG test did statistically improve, however, improved only by 1.2 seconds.

All patients answered positively to the question whether they would undergo the same surgical procedure again if they could go back in time, at 12 months after surgery.

## Discussion

This study aimed to assess functional recovery, patient satisfaction, and the safety of SSBHR in patients with bilateral hip OA. All patients recovered significantly in functionality and QOL within 3 months after SSBHR and 1 year after the surgery. All patients expressed satisfaction by stating their willingness to undergo the same surgical procedure again. In accordance with a systematic review on the safety of SSBHR, we did not observe any major or minor complications, readmissions, or blood transfusions (Shao et al., 2017). The average hospital stay was only 2.9 days, with all patients being discharged directly to their homes.

**Figure 2.**
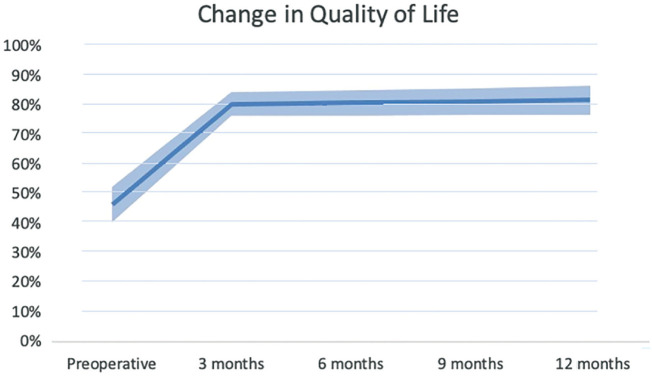
Average recovery courses of health-related quality of life with standard deviation, measured preoperatively and 3 and 12 months after surgery. Interestingly, after a strong improvement, recovery courses flatten 3 months after surgery.

**Figure 3.**
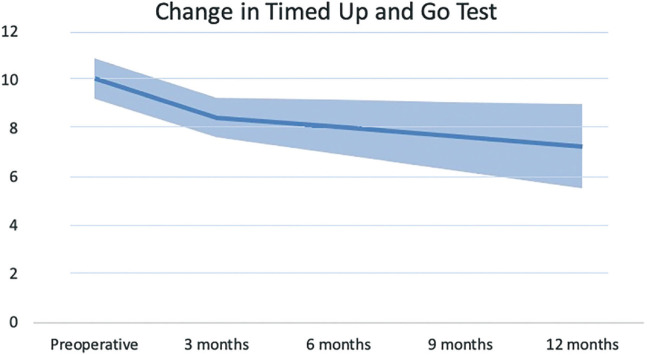
Average recovery courses of the Timed Up and Go test with standard deviation, measured preoperatively and 3 and 12 months after surgery. Interestingly, after a strong improvement, recovery courses flatten 3 months after surgery.

**Figure 4.**
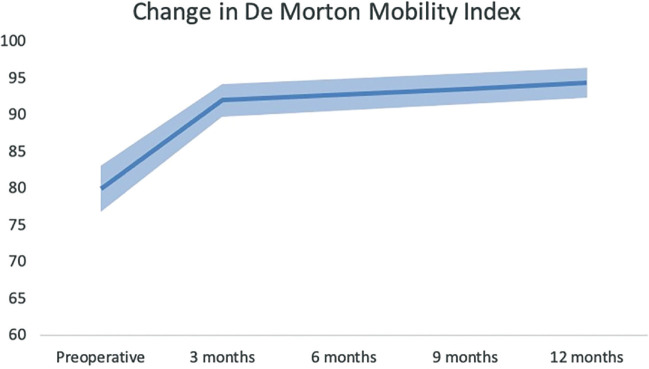
Average recovery courses of the de Morton Mobility Index with standard deviation, measured preoperatively and 3 and 12 months after surgery. Interestingly, after a strong improvement, recovery courses flatten 3 months after surgery.

Our results demonstrate that the most substantial recovery occurs within the first 3 months after surgery. Validated functional tests, including the TUG test and the DEMMI, showed statistically significant improvements above the MCID at 3 months (Alghadir et al., 2015; de Morton et al., 2011; Jans et al., 2011). All SF-36 domain improvements were a great deal better at 3 months than preoperative outcomes (Escobar et al., 2007). Interestingly, the rapid improvement in functional recovery and perceived HRQOL plateau after 3 months (see Figures 2–4). At 12 months postsurgery, both QOL and DEMMI show no statistically significant or clinically important differences compared with the 3-month mark. Although the TUG changes are statistically significant and just within the MCID, the improvement rate decreases considerably (from 0.53 to 0.13 seconds per month on average; see Table 2) (Alghadir et al., 2015). These findings suggest that good functional recovery and significantly improved QOL can be achieved within 3 months after SSBHR.

Our study offers several strengths, including being the first to investigate functional, mental, and vital recovery after SSBHR. Although numerous studies have examined risks and complications associated with the procedure, most are dated and conducted before the fast-track era (Alfaro-Adrián et al., 1999; Parvizi et al., 2006; Shao et al., 2017). Despite the small sample size, our results are statistically significant and clinically relevant. Moreover, our study population appears representative of patients undergoing SSBHR, as it does not differ significantly from other studies included in the systematic review by Shao et al. (2017).

However, our study has some limitations. The lack of a control group prevents us from determining whether SSBHR is a superior intervention for all patients with bilateral OA. In addition, only two surgeons performed the SSBHR procedures, which could introduce bias. The timing of our scoring could also be improved by including more measurement points within the first 3 months, potentially revealing a faster recovery time.

Given the new insights into recovery after SSBHR, healthcare professionals can inform patients suggesting that SSBHR is a viable treatment option for patients with bilateral hip osteoarthritis (Elwyn et al., 2012). Also, other hospitals could consider implementing it as standard of care for treating bilateral symptomatic hip OA. As the first study to provide evidence for rapid, satisfactory recovery after SSBHR, further research is needed to confirm our findings. We recommend conducting a larger, multicenter study to ensure greater external validity and minimize biases and confounding factors related to single-center patient care.

In conclusion, our study found that SSBHR offers a rapid recovery time on physical and HRQOL items, with functional status maximized and significantly improved compared with preoperative scores. Therefore, SSBHR could be considered as a treatment option for patients suffering from bilateral Hip OA. To determine whether SSBHR has better recovery outcomes than two-stage bilateral hip replacement, we recommend conducting a multicenter randomized controlled trial with a large sample size and including at least three different orthopaedic surgeons performing surgery in an equivalent manner.
